# Dr. Alfred Mercer

**Published:** 1914-10

**Authors:** 


Dr. Alfred Mercer, Geneva, 1845, died at his home in Syracuse, August 5, aged 93. He was formerly Professor of Minor and Clinical Surgery, later Prof. of State Medicine and Emeri-

---

tus Prof. of Medicine, in Syracuse University. He served on the state board of health for five years and on that of Syracuse for seven years. He had held many positions of honor, including the presidency of the Syracuse Medical Society, the Onondaga County Medical Society, and the Syracuse Academy of Medicine and the vice-presidency of the State Society. He was a member of the British Medical Association as well as a fellow of the A. M. A. He was one of the very oldest physicians of the state.

Note: Geneva Medical College was established in 1836 and in 1872 was merged with the Medical Dept. of Syracuse University. In the early days, it had an influence on the University of Buffalo and through Ford, the anatomist, on the Medical Dept. of the University of Michigan. We have published many obituary notices of its graduates. We would welcome a historic article from one of its alumni.

